# Comment on “Supraventricular Tachycardia Ablation in the Elderly—Characteristics and Outcomes”

**DOI:** 10.1002/joa3.70346

**Published:** 2026-04-20

**Authors:** Umme Roman, Syed Huzaifa Khan

**Affiliations:** ^1^ Nowshera Medical College Nowshera Pakistan; ^2^ Khyber Medical University Peshawar Pakistan

## Abstract

Catheter ablation for supraventricular tachycardia demonstrates high acute procedural success and acceptable immediate safety in selected elderly patients. However, the absence of long‐term follow‐up, substrate diversity, and broader patient inclusion limits conclusions regarding durable clinical effectiveness.

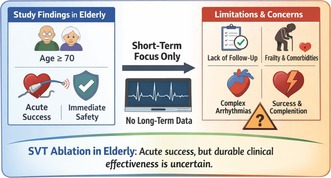

Dear Editor,

Chua et al. published an article entitled “Supraventricular Tachycardia Ablation in the Elderly—Characteristics and Outcomes,” which we read with great interest [1]. The authors examined an important clinical question: How safe and effective is catheter ablation for supraventricular tachycardia (SVT) in elderly patients (defined as ≥ 70 years in the original study) compared with younger patients? In a single‐center, observational, retrospective analysis, a total of 1758 patients were studied, demonstrating that at a high‐volume tertiary center, acute procedural success and immediate complication rates in elderly patients are comparable to those of younger patients. All patients undergoing atrioventricular nodal re‐entry tachycardia (AVNRT), atrioventricular re‐entry tachycardia (AVRT), and/or atrial tachycardia (AT) ablation between May 2011 and May 2022 at a tertiary center were included. This research holds particular significance, especially in the context of expanding use of catheter ablation in elderly patients, where interpretation of acute outcomes may substantially influence clinical decision‐making. However, several important limitations restrict the clinical generalizability of the findings.

First, the study's conclusion that SVT ablation is “safe and effective” in elderly patients relies primarily on acute procedural success and periprocedural complication rates. Although these measures indicate procedural feasibility, the absence of recurrence data, late conduction outcomes, or post‐discharge follow‐up limits interpretation of durable clinical effectiveness, particularly in older patients [2, 3]. Acute non‐inducibility in the electrophysiology laboratory does not capture arrhythmia recurrence, late conduction system disease, symptom burden, functional recovery, or quality‐of‐life outcomes endpoints that are particularly important in elderly populations [4]. Consequently, the reported efficacy should be interpreted as reflecting immediate procedural outcomes, and caution is warranted when extrapolating these findings to long‐term clinical benefit. Furthermore, as only patients who proceeded to ablation were included, the elderly cohort likely represents a pre‐selected population deemed suitable for intervention. Patients with advanced frailty, significant comorbidity burden, or perceived procedural risk may not have been referred or accepted for ablation. This procedural selection may underestimate risk and limit generalizability to the broader elderly SVT population.

Second, the elderly cohort was overwhelmingly dominated by atrioventricular nodal re‐entrant tachycardia and right‐sided ablations, which are well known to carry low complication risk compared with left‐sided pathways or more complex tachycardias [5, 6]. Thus, the favorable safety profile observed may reflect substrate selection rather than age‐independent procedural tolerance [7]. This may limit the external validity of findings for elderly patients with more complex or left‐sided arrhythmic substrates.

Finally, only one complication occurred among 150 elderly patients. With such a low event rate, the study is statistically underpowered to exclude modest but clinically meaningful differences in complication risk. The absence of statistical significance should not be conflated with clinical or practical equivalence [8].

This study provides important evidence that catheter ablation for supraventricular tachycardia achieves high acute procedural success and acceptable immediate safety in carefully selected elderly patients, particularly those with low‐risk arrhythmia substrates. However, the findings primarily demonstrate technical feasibility rather than durable clinical effectiveness. Long‐term follow‐up, substrate‐stratified analyses, and assessment of broader elderly populations are necessary before extending age‐independent procedural recommendations.

Sincerely,

## Funding

The authors have nothing to report.

## Ethics Statement

The authors have nothing to report.

## Consent

The authors have nothing to report.

## Conflicts of Interest

The authors declare no conflicts of interest.

## Data Availability

Data sharing not applicable to this article as no datasets were generated or analysed during the current study.
